# Supplementary material for: Imbalanced IL-1B and IL-18 Expression in Sézary Syndrome
Source: Int J Mol Sci. 2023 Feb 28;24(5):4674. doi: 10.3390/ijms24054674 (PMC10003479; doi:10.3390/ijms24054674)
Supplement: Supplementary file 1 [file ijms-24-04674-s001.zip › ijms-2213748-supplementary.pdf]

Supplementary material

Supplementary Table S1. Sequences of the primers used for qRT-PCR		
Gene	Forward sequence	Reverse sequence
NLRP1	5'AAGACCAGCTGTTCTCGGAGTT3'	5'AGGCATGAGATCTCCTGGTTTC3'
NLRP3	5'TGGAGTGTCGGAGAAGAG3'_	5'TGCTGTCATTGTCCTGGTGT3'
NLRP4	5'GAACTACGGCTGGACTCTTTT3'	5'AGCAAAGACATGTGGCGGAA3'
AIM-2	5'CACCAAAAGTCTCTCCTCATGTT3'	5'AAACCCTTCTCTGATAGATTCCTG3'
IL18	5'GACGCATGCCCTCAATCC3'	5'CTAGAGCGCAATGGTGCAATC3'
IL1B	5'TCCCCAGCCCTTTTGTTGA3'	5'TTAGAACCAAATGTGGCCGTG3'
CARD8	5'CAGTGTCCAAGCAACAAGCC3'	5'TACTGGATCCACTGTCCCGT3'
GAPDH	5'GAAGGTGAAGGTCGGAGT3'	5'GAAGATGGTGATGGGATTTC 3'

Supplementary Table S2. Subjects characteristics

Patient SS	Gender	Age	Lymphocytes	CD4+	CD4+CD26-	CD4+CD7-	CD8+	CD4/CD8	Skin biopsy*	Serum	PBMC	LN biopsy*
SS1	F	36	3620	2908	1192 (41)	930 (32)	263	11	-	X	-	-
SS2	M	62	6870	6270	3762 (60)	2696 (43)	452	11	-	X	-	X
SS3	M	58	5687	4866	2335 (48)	1557 (37)	422	9	-	X	-	-
SS4	F	69	4730	4209	2946 (70)	2147 (51)	94	44	X	X	-	X
SS5	M	62	2100	1008	332 (33)	63 (3)	504	4	-	X	X	-
SS6	F	81	2280	1940	1105 (57)	427 (22)	161	12	X	-	-	X
SS7	M	53	1430	600	540 (9)	222 (37)	328	2	X	-	-	X
SS8	F	76	1160	916	540 (59)	183 (20)	81	11	X	-	-	-
SS9	M	57	3490	3176	2350 (74)	2604 (82)	174	18	-	X	-	X
SS10	M	62	8670	8150	4238 (52)	3341 (41)	347	23	-	X	X	-
SS11	F	60	131010	5767	120736 (96)	120736 (96)	5240	24	X	-	X	-
SS12	M	48	21430	0572	4526 (22)	4114 (2)	428	50	-	X	-	-
SS13	M	68	6830	6251	5376 (86)	5063 (81)	295	21	X	-	-	-
SS14	F	53	3090	2472	1508 (61)	490 (2)	247	10	X	X	X	-
SS15	M	58	7640	6647	5251 (79)	1994 (30)	289	23	-	X	-	-
SS16	F	61	2030	2030	1705 (84)	1218 (60)	182	11	-	X	-	-
SS17	M	48	7640	6647	5251 (79)	2791 (42)	153	43	X	-	-	-
SS18	F	70	7370	6706	5968 (89)	4158 (62)	147	45	X	X	X	-
SS19	F	69	3260	2543	2060 (81)	585 (23)	242	10	X	X	X	X
SS20	M	55	2240	1705	290 (17)	937 (55)	313	6	X	X	-	X
SS21	F	65	3700	3293	2700 (82)	231 (7)	148	22	X	X	X	X
SS22	M	84	2610	2531	2453 (94)	760 (30)	224	11	X	X	X	X
SS23	M	70	2200	2068	1861 (90)	827 (40)	66	31	X	X	X	-
SS24	F	43	5690	4840	4210 (87)	2904 (60)	178	27	-	X	-	-
SS25	M	61	2250	1786	1327 (59)	1417 (63)	98	18	X	X	X	X
SS26	F	40	3780	3080	215 (7)	92 (3)	296	10	-	-	X	-
SS27	M	62	2700	2200	1980 (90)	880(40)	180	12	-	-	-	X
SS28	M	28	1430	600	540 (9)	222 (37)	328	2	-	-	-	X
HD:40	18M/22F	54 (30-85)	ND	ND	ND	ND	ND	ND	12	23	8	ND
IE:19	13M/6F	65 (32-77)	ND	ND	ND	ND	ND	ND	12	ND	ND	8

F= female, M= male; ND= not done; LN= lymph node; *included in the immunohistochemistry analysis;
Reference value for: Lymph (1000-3200/mm3); CD4+ (900-3400/mm3); CD4+CD26 (>40%); CD4+CD7- (>30%.); CD8+(300-1000/mm3); CD4/CD8 ratio (>10%);

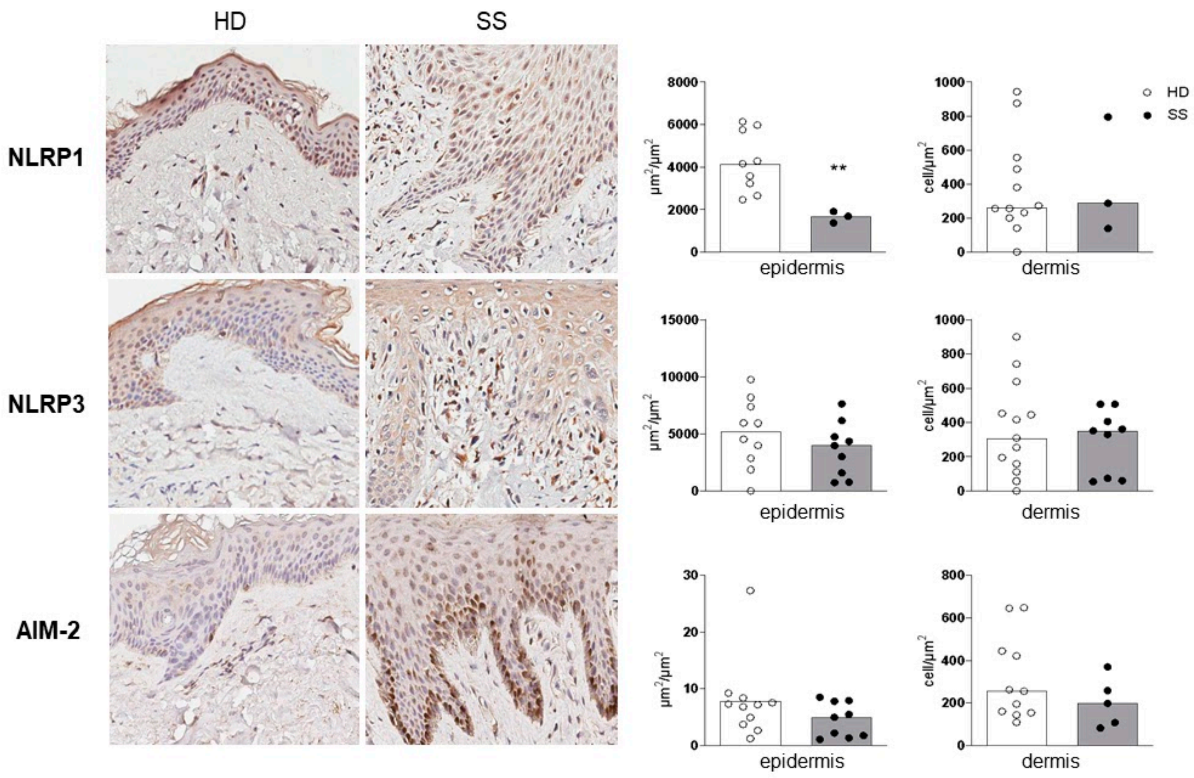

Supplementary Figure S1. Expression of NLRP1, NLRP3 and AIM2 in the skin of SS patients. Expression of NLRP1, NLRP3 and AIM2 were assessed in the skin biopsies of SS patients (n = 3-9, closed circle), healthy individuals (n = 9-13 open circle) by immunohistochemistry. Analyses were performed in the epidermis and dermis. Values are expressed as median. **p≤0.01 compared to HD group.

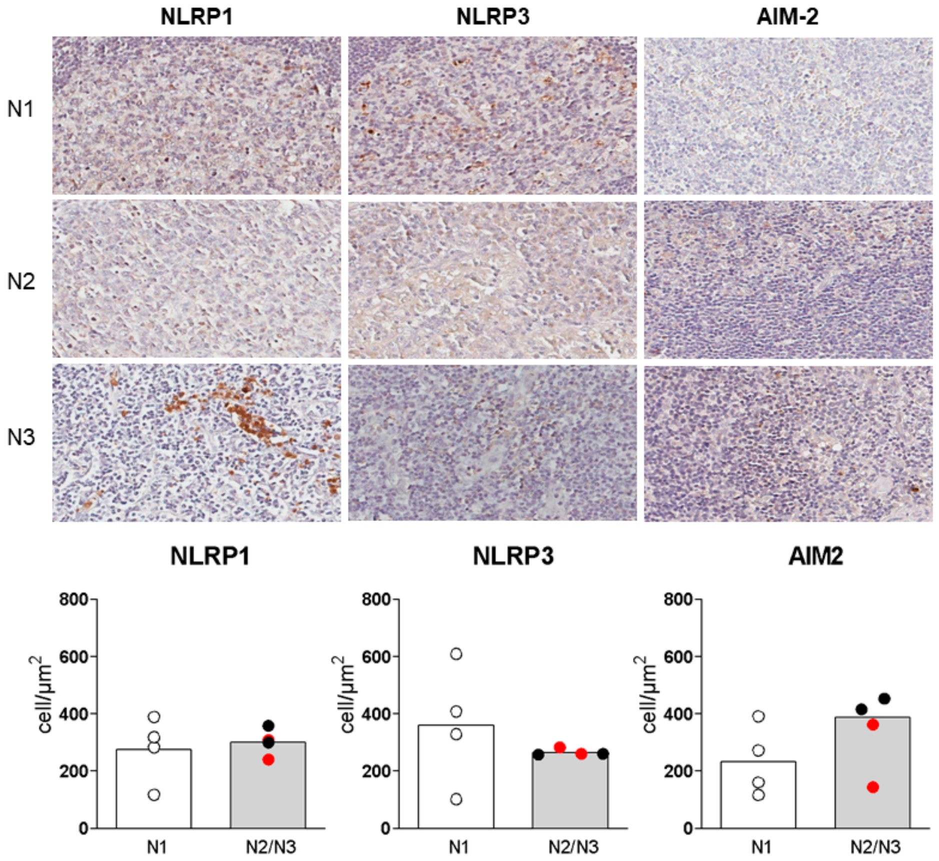

Supplementary Figure S2. Expression of NLRP1, NLRP3 and AIM2 in lymph nodes of SS patients. Expression of NLRP1, NLRP3 and AIM2 were assessed in the lymph nodes biopsies of SS patients at stages N1 (n = 4, open circle), N2 (n = 2, pink circle) N3 (n = 2, closed circle) by immunohistochemistry.

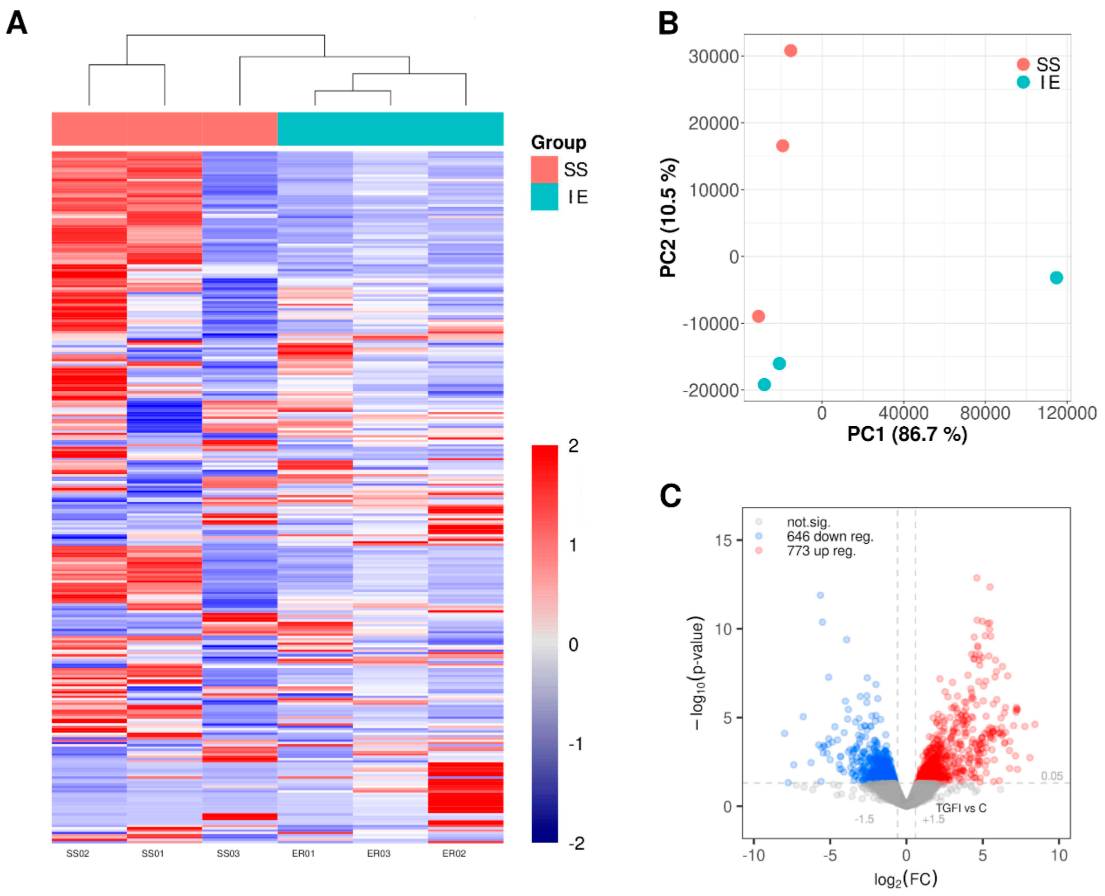

Supplementary Figure S3. Transcriptomic analyses of lymph nodes of SS. A) Unsupervised z-score heatmap of normalized counts of the 300 most variable and expressed genes. The IE group is colored in cyan and the SS group is in pink. Red shades mean expression above the row average, and blue shades means below the average expression. B) Explained variance projection on to the two largest orthogonal principal components. C) Volcano plot of -log10 (p-value) as function of log2FC. Upregulated genes are represented by red cycles and downregulated genes are represented by blue cycles. Gray cycles represent not-significant altered genes. Vertical dashed lines represent absolute FC values limits of 1.5 and the horizontal dashed line represents p-value significance level of 0.05.

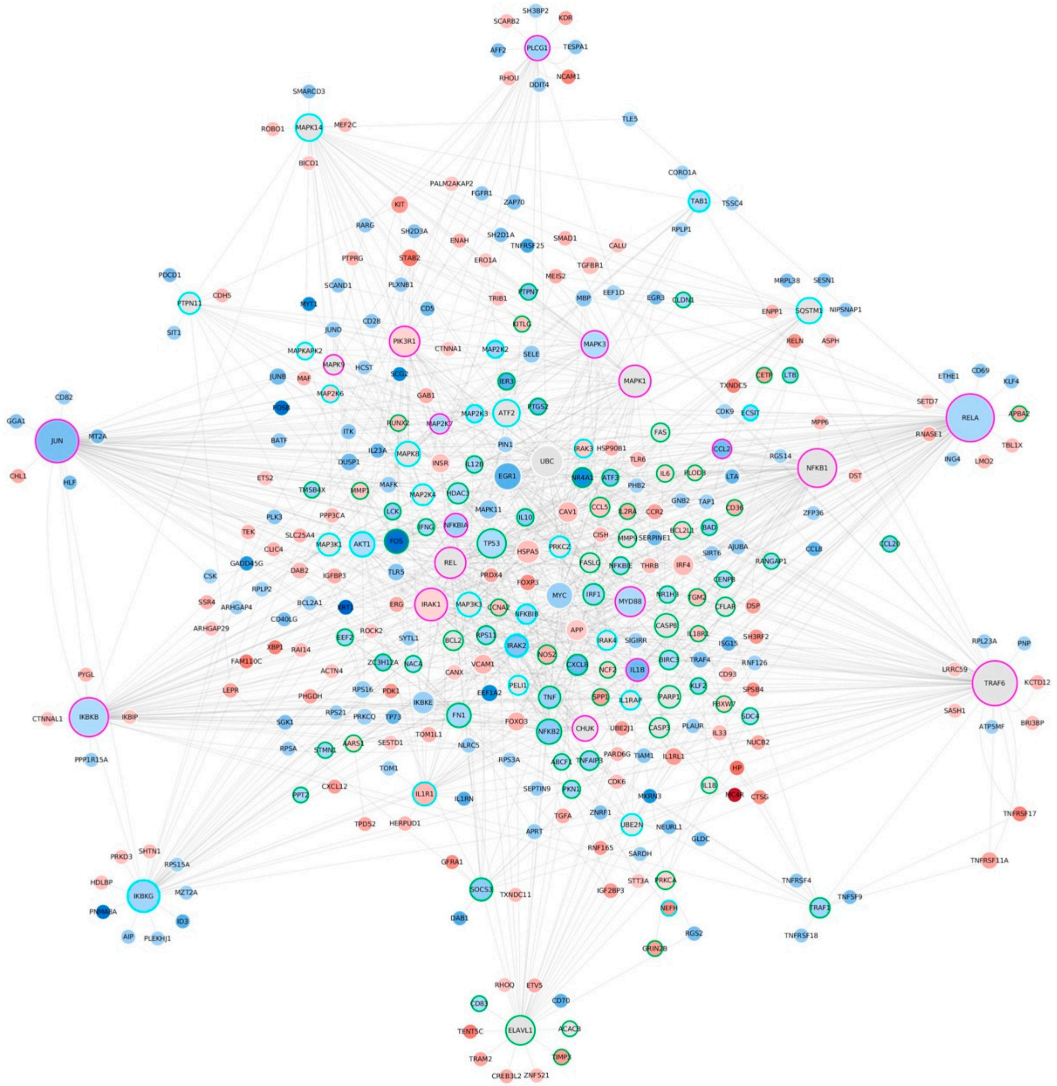

Supplementary Figure S4. Network of the IL-1β and IL-18 signaling pathways genes. Each circle in the network represents a gene. Genes colored in shades of red have FC >= 1.3, while genes colored in shades of blue have FC <= -1.3, in both cases, regardless of the p-value. Circles with a cyan border represent IL1-beta activation signaling pathway genes from the Wikipathway molecular pathways database. Green borders, representing genes participating in the IL18 activation pathway; and genes with the magenta border act in both pathways. The size of each circle is proportional to the number of genes it connects to.
